# Striatal parvalbuminergic neurons are lost in Huntington's disease: implications for dystonia

**DOI:** 10.1002/mds.25624

**Published:** 2013-09-03

**Authors:** Anton Reiner, Evan Shelby, Hongbing Wang, Zena DeMarch, Yunping Deng, Natalie Hart Guley, Virginia Hogg, Richard Roxburgh, Lynette J Tippett, Henry J Waldvogel, Richard LM Faull

**Affiliations:** 1 Department of Anatomy and Neurobiology, The University of Tennessee Health Science CenterMemphis, Tennessee, USA; 2 Centre for Brain Research, University of AucklandAuckland, New Zealand; 3 Department of Psychology, University of AucklandAuckland, New Zealand; 4 Department of Neurology, Auckland City HospitalAuckland, New Zealand; 5 Department of Anatomy with Radiology, University of AucklandAuckland, New Zealand

**Keywords:** Huntington's disease, dystonia, striatum, parvalbuminergic interneurons

## Abstract

Although dystonia represents a major source of motor disability in Huntington's disease (HD), its pathophysiology remains unknown. Because recent animal studies indicate that loss of parvalbuminergic (PARV+) striatal interneurons can cause dystonia, we investigated if loss of PARV+ striatal interneurons occurs during human HD progression, and thus might contribute to dystonia in HD. We used immunolabeling to detect PARV+ interneurons in fixed sections, and corrected for disease-related striatal atrophy by expressing PARV+ interneuron counts in ratio to interneurons co-containing somatostatin and neuropeptide Y (whose numbers are unaffected in HD). At all symptomatic HD grades, PARV+ interneurons were reduced to less than 26% of normal abundance in rostral caudate. In putamen rostral to the level of globus pallidus, loss of PARV+ interneurons was more gradual, not dropping off to less than 20% of control until grade 2. Loss of PARV+ interneurons was even more gradual in motor putamen at globus pallidus levels, with no loss at grade 1, and steady grade-wise decline thereafter. A large decrease in striatal PARV+ interneurons, thus, occurs in HD with advancing disease grade, with regional variation in the loss per grade. Given the findings of animal studies and the grade-wise loss of PARV+ striatal interneurons in motor striatum in parallel with the grade-wise appearance and worsening of dystonia, our results raise the possibility that loss of PARV+ striatal interneurons is a contributor to dystonia in HD.

Motor disturbances represent a disabling and defining feature of Huntington's disease (HD).^1^–^2^ Two disturbances receive the greatest attention—the early occurring chorea/hyperkinesia and the late occurring bradykinesia/akinesia.^1^ The chorea has been attributed to the early preferential loss of enkephalinergic (ENK+) indirect pathway striatal projection neurons projecting to the external pallidal segment (GPe), while the bradykinesia/akinesia has been attributed to the more slowly occurring loss of substance P–containing (SP+) direct pathway striatal projection neurons projecting to the internal pallidal segment (GPi).^3^–^7^ The dystonia that invariably develops by HD grade 2 to 3 is, however, also a significant contributor to HD-related disability and functional decline.^8^,^9^ In juvenile HD and young adult-onset HD, dystonia is, in fact, the presenting and predominant symptom.^8^,^9^

The standard direct-indirect pathway model of basal ganglia function leaves uncertain the basis of the dystonia in HD.^3^,^11^ Considerable recent attention has been devoted to the feed-forward inhibitory influence of parvalbuminergic (PARV+) interneurons on striatal projection neurons,^13^–^19^ and evidence from rodent models suggests that their loss or hypofunction can cause dystonia.^20^–^26^ Thus, the effect of HD on PARV+ striatal interneurons is of interest for understanding dystonia in HD. Existing published data are, however, equivocal as to whether PARV+ interneurons are lost from striatum as HD progresses.^27^,^28^ In the present study, we report that PARV+ striatal interneurons are rapidly lost in HD, and that the progression of loss from motor striatum coincides with dystonia emergence.

## Materials and Methods

### Approach

To quantify PARV+ neuron loss in HD, we determined PARV+ interneuron abundance in caudate and putamen of normal humans, and in HD victims spanning all 4 symptomatic grades. We also counted interneurons co-containing neuropeptide Y-containing (NPY) and somatostatin (SS), and expressed PARV+ interneuron abundance as the ratio of PARV+ interneurons to interneurons co-containing NPY and SS. Since NPY/SS+ interneurons are not lost in HD,^4^–^30^ a decline in the ratio of PARV+ neurons to NPY/SS neurons linearly reflects PARV+ interneuron loss, by controlling for the impact of striatal shrinkage due to advancing HD grade on neuronal spatial density.

### Subjects and Tissues

Coronal tissue blocks or slide-mounted sections containing caudate and putamen at a level rostral to globus pallidus, and/or at a level posterior to the anterior commissure at which GPe and GPi are well formed (and henceforth called mid-putamen or motor putamen) were obtained for 37 HD cases (male = 18; female = 15; unknown = 3) verified by pathology, symptoms, family history, and/or CAG repeat, with age at death ranging from 35 to 87 years (mean age = 60.7 ± 2.2) (Table1). Three were obtained from the University of Michigan Medical Center (Ann Arbor, MI, USA), 3 from the National Neurological Resource Bank (NNRB, Los Angeles, CA, USA), 2 from the Harvard Brain Tissue Resource Center (HBTRC, Belmont, MA, USA), 2 from the Douglas Hospital Research Center (DHRC, Montreal, Quebec, Canada), 2 from the University of Rochester (UR, Rochester, NY, USA), and 25 from the Neurological Foundation of New Zealand Human Brain Bank (Auckland, New Zealand). The mean death age for the HD cases was generally less with advancing grade (Table1), which reflects the tendency of disease severity to be associated with earlier death.^31^ Mean CAG repeat for the HD allele also increased with HD grade, ranging from 41.2 for grade 1 cases to 50.0 for grade 4 cases. This is consistent with prior findings that higher CAG repeats are associated with greater disease severity.^31^

**Table 1:** Tabular grade-wise listing of information about the human cases examined here, including gender, age at death, PMD between death and brain fixation, CAG repeat, and the overall number of cases and the case per striatal region

Grade	Males (n)	Females (n)	Gender not known (n)	Total cases (n)	Age (years)	PMD (hours)	WT allele CAG	HD allele CAG	Cases with rostral caudate (n)	Cases with rostral putamen (n)	Cases with mid-putamen (n)
Control	15	6	4	25	60.3	13.8	17.0	20.6	19	19	14
1	5	2	2	9	64.6	9.8	17.8	41.2	5	6	4
2	5	5	0	10	63.5	12.6	20.3	42.9	9	8	3
3	6	7	0	13	55.0	14.0	18.6	45.3	8	8	8
4	2	3	0	5	58.4	17.2	18.0	50.0	3	3	3

PMD, postmortem delay; WT, wild-type; HD, Huntington's disease.

Coronal tissue blocks or slide-mounted sections containing caudate and putamen at a rostral basal ganglia and/or a mid-basal ganglia level were also obtained for 25 control specimens from the University of Michigan Medical Center (1), the HBTRC (2), the UR (1), The University of Tennessee Health Science Center (UTHSC) Department of Pathology (2), and the Neurological Foundation of New Zealand Human Brain Bank (19). The control cases included 15 males, 6 females, and 4 cases of unknown gender, with age at death ranging from 15 to 81 (mean = 60.3 ± 3.5) years (Table1). All control specimens were neurologically normal, except for 1 Parkinson's disease case. Control specimens were from individuals whose autopsies were performed within the same range of dates as HD cases.

Brains from all sources other than University of Auckland were obtained at autopsy and immersion-fixed in formalin. Brains from the New Zealand Human Brain Bank were perfused as described previously through the basilar and internal carotid arteries with 15% of formalin in 0.1 M phosphate buffer (pH 7.4) for 1 hour.^32^ Postmortem delay for control brains ranged from 4.5 to 39 (mean = 13.8 ± 1.6) hours, and for HD brains from 4 to 31 (mean = 12.5 ± 1.1) hours (Table1). Differences in age at death and postmortem delay between HD cases and controls were not statistically significant, and HD and control cases were well matched for agonal status. HD cases were staged according to Vonsattel et al.,^33^ and the HD specimens included 9 grade 1, 10 grade 2, 13 grade 3, and 5 grade 4 cases (Table1).

As our goal was to determine if loss of PARV+ striatal interneurons helped explain the pathophysiology of dystonia in HD, information on motor symptoms in our HD cases was pertinent. Clinical information was, however, only available for 2 of our 9 grade 1 cases, 4 of our 10 grade 2 cases, 9 of our 13 grade 3 cases, and 2 of our 5 grade 4 cases, and in many cases was not current with date of death. The limited clinical information indicated, as expected, that the prevalence of dystonia increased with advancing grade, being absent in our grade 1 cases, present in half of the grade 2 cases, and present in all of the grade 3 and 4 cases. Due, however, to the facts that the clinical information was in many cases not current with date of death and was rarely a quantified dystonia score, we were unable to statistically correlate degree of dystonia with magnitude of PARV+ interneuron loss.

### Immunohistochemical Methods

Since our tissue was of diverse types in terms of section thickness and disease grade, we chose to express the abundance of PARV+ neurons in striatum as a ratio of PARV+ neurons counted to interneurons co-containing NPY and/or SS counted, adjusted by the Abercrombie correction for perikarya size. Tissue blocks for HD and/or control cases from DHRC, UR, UM, NNRB, and UTHSC were immunostained for parvalbumin (PARV), NPY, and/or SS at UTSHC using methods described in Deng et al.,^5^–^13^ using antisera whose specificity has been previously shown.^4^–^36^ Tissue from the New Zealand Human Brain Bank was processed by immunolabeling for PARV, NPY, and/or SS at the Centre for Brain Research of the University of Auckland using described procedures.^32^,^37^ Further details are provided in the Supporting Information.

### Quantification of Neuronal Abundance

We counted PARV+ interneurons, NPY+ interneurons, and SS+ interneurons in rostral caudate, rostral putamen, and mid-putamen. After an Abercrombie double-counting correction, the abundance of the PARV+ interneurons, NPY+ interneurons, and SS+ interneurons were expressed per mm^2^ for each region in each case. The abundance of NPY/SS neurons was considered to be the average of the NPY and SS counts in those instances in which both were available. The final NPY/SS neuron count for each case and each striatal region was used to express PARV+ interneuron abundance as a ratio to NPY/SS interneuron abundance. For simplicity, we will refer to the PARV+ interneuron to NPY/SS interneuron ratio as the PARV/NPY ratio in the Results, as in most instances only NPY immunolabeling was available. One-way analysis of variance (ANOVA) with post hoc analysis (Fischer LSD) was used to evaluate results. Further details on our neuron counting methods are provided in the Supporting Information.

## Results

**Figure 1:**
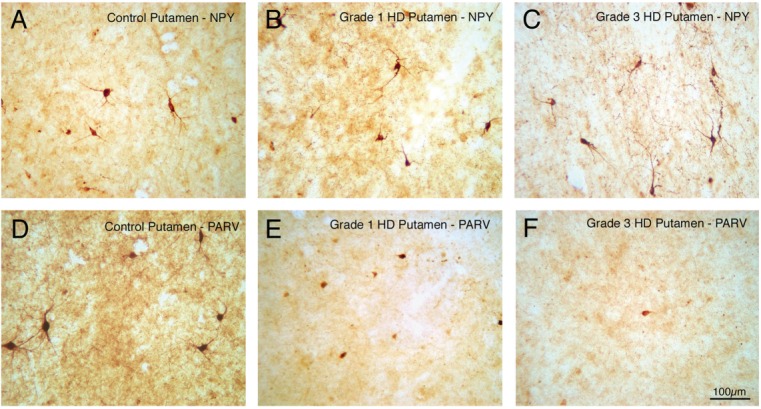
Series of images showing NPY+ striatal interneurons (A-C) and PARV+ striatal interneurons (D-F) in putamen of a control case (A, D), grade 1 HD case (B, E), and grade 3 HD case (C, F). Note that NPY neurons show no alteration in size, abundance, or labeling intensity with HD progression, while PARV+ interneurons show progressive decline in all 3 parameters. Magnification is the same in all images. NPY, neuropeptide Y; PARV, parvalbumin; PARV+, parvalbuminergic; HD, Huntington's disease.

Examination of our control and HD cases suggested that a large decrease in PARV+ interneurons occurs in striatum with increasing pathological disease grade. In these same cases, the NPY+/SS+ striatal interneurons were preserved, as expected based on prior reports.^4^ PARV+ interneurons were progressively fewer with advancing HD grade, and they also appeared to be diminished in size, perikaryal labeling intensity, and dendritic labeling. The abundance of PARV+ interneurons in grade 1 HD mid-putamen was not notably different from that in control putamen, although the labeling intensity of neurons appeared reduced. By contrast, PARV+ interneuron abundance in caudate at all grades and in putamen at grades 2 to 4 was clearly reduced. Examples of immunolabeling for PARV+ and NPY/SS+ interneurons in control, grade 1 HD, and grade 3 HD are shown in Figure 1.

**Figure 2:**
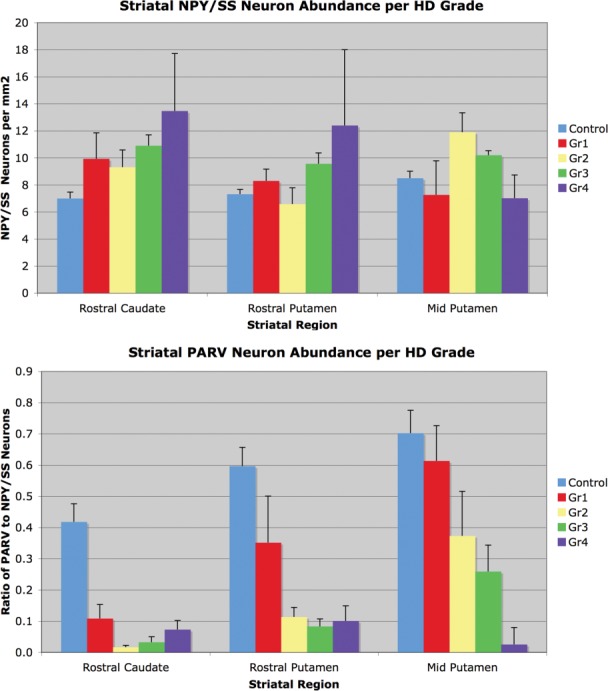
Graphs showing the mean abundance (±SEM) of NPY/SS interneurons across control and HD symptomatic grades in rostral caudate, rostral putamen, and mid-putamen (A), and the mean abundance (±SEM) of PARV+ interneurons across control and HD symptomatic grades for rostral caudate, rostral putamen, and mid-putamen (B). No decline occurs for NPY/SS interneurons, but PARV+ interneurons show prominent loss in HD that is greatest for rostral caudate, and more gradual for mid-putamen. NPY, neuropeptide Y; SS, somatostatin; HD, Huntington's disease; SEM, standard error of the mean; PARV, parvalbumin; PARV+, parvalbuminergic.

Quantitative analysis confirmed and extended these observations. As shown in Figure 2A, NPY/SS+ neuron abundance showed a trend toward an increase in spatial density across advancing HD grades, although the differences were only significant between control and grade 4 for rostral striatum. Note that because of the relatively few grade 4 cases for each region, the grade 4 NPY/SS+ neuron counts tended to be variable, but still significantly more than in control in the case of the rostral caudate and putamen.

The PARV/NPY ratios revealed a profound and highly statistically significant loss (*P* < 0.0077) of PARV+ neurons in the rostral caudate nucleus at all grades, with an approximately 75% loss evident already at grade 1 (Fig. 2B). The grade 1 loss of PARV+ interneurons was less prominent for the rostral putamen than for the rostral caudate, but significant nonetheless (*P* < 0.0120). The losses at HD grades 2 to 4 for rostral putamen were substantial (>80%), and the difference between control and HD grades 2 to 4 was highly significant (*P* < 0.0001). In the case of mid-putamen, PARV+ neuron abundance was indistinguishable from control at grade 1, but then significantly and progressively less than control over grades 2 to 4 (Fig. 2B). Table2 shows the loss of PARV+ interneurons per grade and per region expressed as a percent of control for that region. Note also that NPY/SS+ interneuron abundance in control cases was similar for all 3 striatal regions examined (Fig. 2A), while PARV+ interneuron abundance was significantly greater in mid-putamen than rostral caudate (*P* < 0.0001), and intermediate in rostral putamen (Fig. 2B).

**Table 2:** Tabulation of PARV+ striatal interneuron abundance in rostral caudate and putamen, and mid-putamen, for controls and each HD grade

	PARV neuron abundance in rostral caudate (%)	PARV neuron abundance in rostral putamen (%)	PARV neuron abundance in mid-putamen (%)
Control	100.0	100.0	100.0
HD grade 1	25.9	59.0	87.3
HD grade 2	3.9	19.1	53.1
HD grade 3	7.8	13.9	36.8
HD grade 4	17.5	16.8	3.6

Results are expressed as a percent of control per region, and the PARV interneuron abundance is based on the ratio of PARV+ interneurons to NPY/SS interneurons.

PAV, parvalbmin; PARV+, parvalbuminergic; HD, Huntington's disease; NPY, neuropeptide Y; SS, somatostatin.

## Discussion

Our analysis indicates a large decrease in PARV+ striatal interneurons during HD progression. Even prior to loss, PARV+ interneurons appear to show shrinkage and diminished PARV content, indicative of disease-related dysfunction. As in rodents and nonhuman primates, the fast spiking PARV+ interneurons are twice as abundant in motor putamen as in caudate or more rostral putamen,^13^,^14^ and their loss seems slower in this region than in caudate or more rostral putamen. The loss from motor putamen coincides with the transition from chorea to dystonia in HD,^8^,^9^ with dystonia typically becoming prominent by grade 3 and PARV+ interneuron abundance in motor putamen dropping below 50% after grade 2.

Recent animal studies show that dysfunction of the PARV+ striatal interneurons can yield dystonia. For example, dystonic attacks in *dt^sz^* mutant Syrian hamsters occur during their first 2 months of life, and are associated with abnormalities in striatal projection neuron function attributable to a developmental delay in the maturation of PARV+ interneurons in dorsolateral somatomotor striatum.^20^–^38^ The overall evidence indicates that a transient 20% deficiency in PARV+ interneuron maturation causes spasmodic dystonia due to deficient feed-forward inhibition of SP+ striato-GPi neurons, leading to significantly decreased basal discharge in GPi.^21^–^41^ The finding of normalized PARV+ striatal interneuron abundance and activity in GPi following remission of dystonia further supports the role of the PARV+ interneuron deficiency and the reduced GPi activity in dystonia in the *dt^sz^* hamster.^20^ Similarly, Gittis et al.^26^ reported that infusion of IEM-1460 (an inhibitor of GluA2-lacking 2-amino-3-(3-hydroxy-5-methyl-isoxazol-4-yl)propanoic acid [AMPA] receptors) into mouse sensorimotor striatum, which preferentially blocks excitation of PARV+ interneurons, elicits dystonia. Moreover, haploinsufficiency of the *Nkx2.1* gene in humans causes dystonia,^42^ which may stem from deficient migration of PARV+ neurons from the medial ganglionic eminence into striatum during development. Finally, recording studies in humans show that, like in *dt^sz^* hamsters, GPi neuronal firing is reduced in dystonic individuals.^43^–^47^

**Figure 3:**
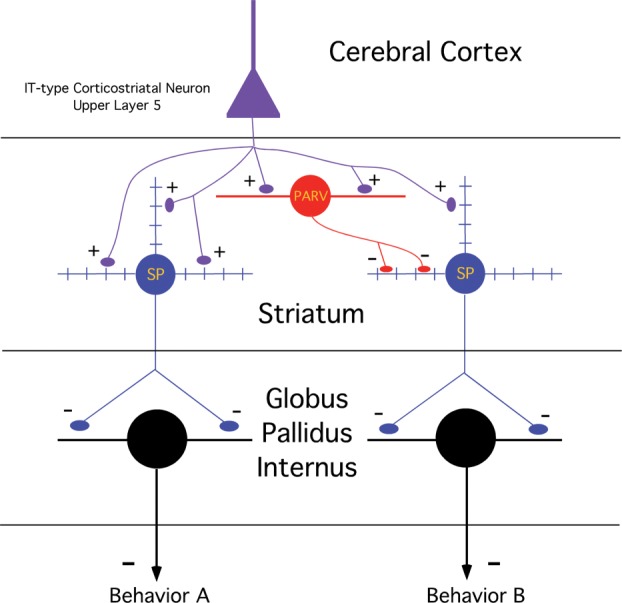
Schematic illustrating hypothesized role of PARV+ striatal interneurons in controlling the responses of nearby SP-containing direct pathway striatal neurons to their driving input from IT-type corticostriatal neurons. Rapid feed-forward inhibition of SP+ neurons by cortically activated PARV+ neurons is hypothesized to suppress the responses of SP+ striatal neurons in nearby domains but not within the domain of the PARV+ interneuron itself. In the illustration, by suppressing cortical activation of SP+ neurons controlling behavior B, the PARV+ interneuron ensures that IT-type cortical input activates SP+ neurons controlling behavior A. In the absence of the PARV+ interneuron, the conflicting behaviors A and B would both be initiated, leading potentially to dystonia if A and B involve opposing muscle groups. PARV, parvalbumin; PARV+, parvalbuminergic; SP, substance P; IT, intratelencephalically projecting.

The anatomy and physiology of PARV+ striatal interneurons is consistent with the idea that their loss can lead to dystonia. PARV+ interneurons fire repetitively when depolarized by cortical stimulation, with a shorter latency and lower threshold than striatal projection neurons.^14^–^48^ As a consequence, cortical activation of PARV+ neurons prevents or reduces the response to this same cortical activation of the striatal projection neurons to which the PARV+ interneurons project.^16^,^19^ PARV+ interneurons have much of their axonal arborization beyond their own dendritic field,^16^ and PARV+ interneurons preferentially inhibit SP+ striato-GPi neurons.^49^–^50^ Given the reported small size of the cortical terminals ending on PARV+ interneurons, it seems likely that they receive their cortical input from the intratelencephalically projecting type (IT-type) corticostriatal neurons, which also preferentially innervate SP+ striatal neurons.^51^–^52^ Thus, SP+ neurons lying within the dendritic field of a given PARV+ neuron would be activated by convergent input from diverse cortical areas, as would be the given PARV+ interneuron itself.^51^,^53^ The SP+ neurons outside the PARV+ interneuron domain, however, also receive input from some of the same IT-type neurons due to the diffuse nature of the IT-type axonal arborization (Fig. 3). As a result, both sets of SP+ neurons can be activated, though to differing degrees, by the same IT-type input, potentially leading to facilitation of conflicting movements.^3^–^11^ If the cortical activation within the domain of a particular PARV+ interneuron exceeds that to other nearby domains, rapid feed-forward inhibition of the SP+ neurons in neighboring domains from this activated PARV+ interneuron may serve to suppress their responses, ensuring that only a narrow set of SP+ neurons is sufficiently activated to trigger a particular movement. Consistent with this interpretation, Gage et al.^55^ found that PARV+ striatal interneurons in behaving rat were active at choice points, suggesting PARV+ interneurons suppress nonpreferred behaviors.

Thus, PARV+ interneurons may act locally^56^ to regulate nearby SP+ striatal neuron activity in response to cortical input. Studies in monkeys have shown that the putamen contains somatotopically organized microexcitable zones from which body movement can be elicited,^57^ supporting the view that cortical activation of striatal projection neuron sets can trigger movement. Moreover, fMRI studies show participation of motor putamen in humans in movement sequencing during task execution.^58^ Given the apparent role of PARV+ interneurons, therefore, it would be predicted that their loss with preservation of SP+ neurons (as occurs in mid-HD) should yield a basal ganglia that simultaneously initiates conflicting motor routines, exhibiting as dystonia. With the extensive loss of enkephalinergic striatal projection neurons by mid-grade HD, it may be that loss of PARV+ interneurons mainly affects SP+ neuron activity during HD progression.

Thus, PARV+ striatal interneuron loss would be expected to contribute to dystonia in HD. Existing published data had, however, been equivocal as to whether PARV+ interneurons are lost from the striatum as HD progresses.^27^–^28^ In the present study, we found that PARV+ striatal interneurons are rapidly lost as HD progresses, especially from caudate. The loss in putamen is more gradual, especially for motor putamen. Nonetheless, the loss even for motor putamen was >50% by grade 3 HD, and PARV+ interneuron shrinkage and PARV loss appeared to occur even before then. In general, the loss of PARV+ striatal interneurons per grade and region seems as prominent as the overall loss of striatal projection neurons.^2^–^33^ The vulnerability of PARV+ striatal interneurons in HD stands in contrast to the resistance of 2 other striatal interneuron types to loss, namely cholinergic interneurons and interneurons co-containing SS, NPY, and/or neuronal nitric oxide synthase.^4^,^30^ A vulnerability of PARV+ interneurons, and a resistance of cholinergic interneurons and somatostatinergic interneurons, however, also is evidenced following transient global ischemic insult to striatum,^36^,^60^ and intrastriatal injection of the NMDA-receptor excitotoxin quinolinic acid.^26^–^35^ The basis of the vulnerability of PARV+ striatal interneurons in HD is uncertain, but given their prominent excitatory input, their enrichment in Ca^2+^-permeable AMPA receptors and their BDNF dependence, excitotoxicity, Ca^2+^-mediated injury, or BDNF deprivation could be candidate pathogenic mechanisms.^13^,^35^

Nonetheless, it is not established that PARV+ striatal interneuron loss explains HD dystonia. Another possible explanation could be that GPi output is diminished, not because of increased striato-GPi neuron activity due to PARV+ interneuron loss, but due to GPi neuron loss, leading to disinhibition of motor thalamus domains controlling antagonistic muscle groups. The observation that GPi hypoactivity occurs in human generalized and segmental dystonia,^44^–^64^ as well as in mouse models of DYT1 human dystonia,^65^ is consistent with this possibility. Additionally, 35% deficiency in PARV+ putamen interneurons has been reported in Tourette syndrome,^66^–^67^ without evidence of dystonia. These studies, however, also found a 2.5-fold elevation in PARV+ neurons in the GPi, and they attributed the GPi excess and putamen shortfall in PARV+ neurons to a possible migration defect during development. Thus, the absence of dystonia despite the deficiency in striatal PARV+ interneurons and the occurrence of Tourette symptoms may stem from an alteration in GPi function stemming from its PARV+ neuron excess. Finally, it is also possible that the extensive loss of SP+ neurons by grade 3 HD plays a role in the observed dystonia, since mutant mice with prenatal or postnatal ablation of D1 dopamine receptor-possessing neurons (mainly SP+ neurons) typically have dystonia as a symptom.^68^,^69^ These results must be viewed with caution, however, since ablation of neurons possessing D1 receptors also eliminates D1 receptor-bearing cortical neurons. Thus, it is uncertain if the dystonic phenotype in these mice is attributable to loss of SP+ striatal neurons, or whether loss of cortical D1 neurons accounts for the phenotype.^71^ In any event, the present findings show that PARV+ striatal interneuron loss is prominent in HD, and available animal data on basal ganglia function are consistent with the view that this loss in motor striatum might contribute to dystonic symptoms. Further studies are, however, needed to evaluate the possible contributions of GPi neuron loss or striatal SP+ neuron loss to the pathophysiology of dystonia in HD. Similarly, characterization of PARV+ striatal interneuron loss in HD cases with quantitative assessment of dystonia near the time of death, such as possible for HD patients enrolled in the COHORT and Enroll-HD observational studies,^72^–^73^ would aid evaluation of the role of PARV+ striatal interneuron loss in HD, since it would enable correlation between such neuron loss and the degree of dystonia.
