# Supplementary material for: Striatal parvalbuminergic neurons are lost in Huntington's disease: implications for dystonia
Source: Mov Disord. 2013 Sep 3;28(12):1691–9. doi: 10.1002/mds.25624 (PMC3812318; doi:10.1002/mds.25624)
Supplement: Supplementary file 1 [file mds0028-1691-sd1.doc]

**Supporting Information**

**Immunohistochemical Methods.** Tissue blocks for HD and/or control cases from DHRC, UR, UM, NNRB, and UTHSC were transferred to 20% sucrose/10% glycerol/0.1M sodium phosphate buffer (PB) (pH 7.4). Frozen sections were cut at 60µm with a sliding microtome, and the free-floating sections were then processed by immunohistochemistry for PARV, NPY and/or SS. Specimens from the HBRTC had been sectioned with a cryostat at 7µm, thaw-mounted onto gelatin-coated slides, and shipped to us frozen by overnight mail. These sections were immunolabeled on-the-slide for PARV, NPY and/or SS. To enhance immunolabeling for PARV, NPY and/or SS in tissue processed at UTSHC, sections were immersed for 30 min in 1% sodium borohydride in PB, 0.1 M DL-lysine in PB, followed by 0.2% nonfat dry milk in PB. Basal ganglia blocks from the New Zealand Human Brain Bank were sectioned at 50 µm on a freezing microtome, and the sections were processed free-floating by immunolabeling for PARV, NPY and/or SS. Tissue from this source was processed at the Centre for Brain Research of the University of Auckland, while all other tissue was processed at UTHSC. University of Auckland immunolabeling procedures are described in Thu et al.,^64^ while those used at UTHSC are described in Deng et al.^11,12^

Sections processed at University of Auckland were washed (3x 15 min) in sodium phosphate buffered saline and 0.2% Triton-X (sodium phosphate buffered saline-triton X-100), incubated for 20 min in 50% methanol and 1% H2O2, washed, and incubated in primary antibody for 3 days on a shaker at 4**°**C. The primary antibodies used were mouse anti-PARV at 1:5000 (SWANT or Sigma, clone 235), rabbit anti-NPY at 1:3000 (Amersham #RPN1702), or at 1:8000 (Sigma #9528), and rabbit anti-SS at 1:2000 (Watpa Enterprises, Auckland NZ).^69^ Subsequent to primary antibody incubation, sections were washed, incubated overnight in biotinylated goat anti-mouse secondary antibody (Sigma, 1:500), and then washed, incubated for 4 h at room temperature in ExtrAvidinTM (Sigma, 1:1000), washed, then exposed to 0.05% 3,3-diaminobenzidine tetrahydrochloride (DAB) (Sigma) and 0.01% H_2_O_2_ for 15–20 min to produce a brown reaction product. The sections were washed, mounted on gelatin–chrome–alum coated slides, dried, dehydrated through a graded alcohol series to xylene and coverslipped with Hystomount (Hughes and Hughes, UK). The primary antibodies used for tissue run at UTHSC were mouse anti-PARV at 1:1000 (SWANT or Sigma), sheep anti-NPY at 1:500 (gift from W. Blessing and J. Oliver, Flinders University, Flinders Australia) or rabbit polyclonal anti-NPY from ImmunoStar at 1:500, and mouse anti-SS at 1:500 (gift from Drs. J.C. Brown and S.R. Vincent, UBC, Vancouver, Canada) or rabbit polyclonal anti-SS from ImmunoStar. These antisera are specific and they effectively immunolabel postmortem-fixed human tissue. ^3,9,19,48^ Immunolabeling was carried out as previously.^11,12^ Slide-mounted sections were incubated in primary antisera droplets overnight in humidified chambers, while free-floating sliding microtome sections were incubated in microcentrifuge vials for 48–72 h at 4°C. After primary antibody incubation, sections were processed by the peroxidase-antiperoxidase method, followed by visualization with DAB. Sections were mounted onto gelatin-coated slides, dried, cleared and coverslipped with Permount®.

**Quantification of Neuronal Abundance**. We separately analyzed caudate at a level rostral to globus pallidus, putamen at this same level, and putamen at the level of globus pallidus. Caudate was typically small at the level of globus pallidus and was not analyzed. Caudate or putamen were outlined, and a grid drawn to divide each into a series of approximately 2mm tall zones. Five randomly selected images were captured using a 40x objective for each zone in caudate and/or putamen in each section, and counted by either of two blinded observers for the PARV+ interneurons, NPY+ interneurons, and SS+ interneurons. A mean for each case and each region (rostral caudate, rostral putamen, and mid putamen) was then calculated from the individual measures. After double-counting correction using neuron size and section thickness according to the Abercrombie method, the abundance of the PARV+ interneurons, NPY+ interneurons, and SS+ interneurons was expressed per mm^2^ for each region in each case. For neuron diameter, 10 neurons in rostral putamen from each of two or three control and grade 1-3 cases, and one grade 4 case were measured. In general, NPY+ neurons showed no change in diameter with disease grade, but PARV+ neuron diameter was halved by grade 4. The abundance of NPY/SS neurons was considered to be the average of the NPY and SS counts in those instances in which both were available. Otherwise either the SS or NPY count was used. In those cases in which we had both NPY and SS counts, they were extremely similar. Because the immunolabeling performed at UTHSC was seemingly more sensitive and yielded more labeled neuronal perikarya, the counts of UTHSC-processed tissue were standardized to the counts for New Zealand tissue. To achieve this, a correction factor was applied for the PARV counts and another for the NPY/SS counts, so that the control UTHSC means matched the control New Zealand means for PARV and NPY/SS counts. These same correction factors were also used for the neuronal counts in HD tissue processed at UTHSC. Separate correction factors were used for caudate, rostral putamen, and mid-putamen. The final NPY/SS neuron count for each case and each striatal region was used to express PARV+ interneuron abundance as a ratio to NPY/SS interneuron abundance. Note that in most cases, we only had either NPY+ or SS+ interneuron counts, and most commonly NPY alone. For this reason and for simplicity, we will refer to the PARV+ interneuron to NPY/SS interneuron ratio as the PARV/NPY ratio in the Results. One-way ANOVA with post-hoc analysis (Fischer LSD) was used to evaluate differences in PARV/NPY per target area between controls and the different HD grades for each region analyzed. We additionally evaluated NPY/SS neuron abundance across controls and HD grades by one-way ANOVA with post-hoc analysis (Fischer LSD).
